# CT-guided radiofrequency ablation of facial and mandibular nerves in the treatment of compound Meige’s syndrome

**DOI:** 10.1007/s00234-024-03392-1

**Published:** 2024-06-07

**Authors:** Hao Huang, Bing Huang, Xindan Du, Huidan Lin, Xue Li, Xian Zhao, Qinghe Zhou, Ming Yao

**Affiliations:** 1 Department of Pain Medicine, The Second Affiliated Hospital of Zhejiang University, Hangzhou, 310000 China; 2 Department of Pain Medicine, The Affiliated Hospital of Jiaxing University, Jiaxing, 314000 China; 3 Department of Pain Medicine, The Redcross Hospital of Hangzhou, Hangzhou, 310003 China; 4 Department of Pain Medicine, The Affiliated Hospital of Ninbo University, Ningbo, 315000 China; 5 Department of Pain Medicine, ShuLan Hangzhou Hospital, Hangzhou, 310000 China

**Keywords:** Meige’s syndrome, Radiofrequency ablation, Computed tomography

## Abstract

This retrospective study examined the clinical outcomes and complications in 6 cases of compound Meige’s syndrome, presenting with blepharospasm and masticatory muscle spasm, following treatment with CT-guided radiofrequency ablation targeting bilateral facial nerves and mandibular branches of the trigeminal nerve. After the operation, the symptoms of eyelid spasm and masticatory muscle spasm resolved, but mild facial paralysis and numbness of mandibular nerve innervation persisted. Follow-up for 4–28 months showed that the symptoms of facial paralysis resolved within 2–5 (3.17 ± 0.94) months after the operation, whereas the numbness in the mandibular region persisted, accompanied by a decrease in masticatory function. During the follow-up period, none of the 6 patients experienced a recurrence of Meige’s syndrome. These findings suggest that CT-guided radiofrequency ablation of the bilateral facial nerve and mandibular branches of the trigeminal nerve may offer a promising approach to treating compound Meige’s syndrome.

**Supplementary Information:**

The online version contains supplementary material available at 10.1007/s00234-024-03392-1.

## Introduction

Meige’s syndrome is characterized by bilateral blepharospasm and/or masticatory muscle spasm symmetrically distributed along the central axis of the face [1], which belongs to segmental craniocervical dystonia [2]. The combination of blepharospasm and masticatory muscle spasm is called compound Meige’s syndrome. Local injection of botulinum toxin can only provide relief from the symptoms in blepharospasmic Meige’s syndrome for approximately 3 months [3]. Although deep brain stimulation (DBS) can offer benefits to 60–70% of patients with Meige’s syndrome [4], the treatment costs, as high as US $20,000, often make it unaffordable for patients without insurance coverage in China. Based on facial nerve radiofrequency therapy for hemifacial spasms, we have demonstrated the efficacy of bilateral facial nerve radiofrequency in treating blepharospasmic type Meige’s syndrome [5]. In this stduy, we reported the clinical outcomes of CT-guided combined radiofrequency ablation targeting both the facial nerve and the mandibular branch of the trigeminal nerve for compound Meige’s syndrome.

## Methods

This retrospective study included patients diagnosed with Meige’s syndrome from April 2021 to March 2023. Patients meeting the following inclusion criteria were enrolled in this study: (1) diagnosis of Meige’s syndrome, specifically the type characterized by blepharospasm combined with masticatory muscle spasm [1]; (2)who accepted CT-guided radiofrequency ablation. Patients with any of the following conditions were excluded from the study: (1)just accepted radiofrequency ablation of the facial nerve; (2)just accepted radiofrequency ablation of the mandibular branch of the trigeminal nerve. This study was approved by the Ethics Committee of the Affiliated Hospital (approval number: LS2019-013), and all patients signed an informed consent form.

**Fig. 1:**
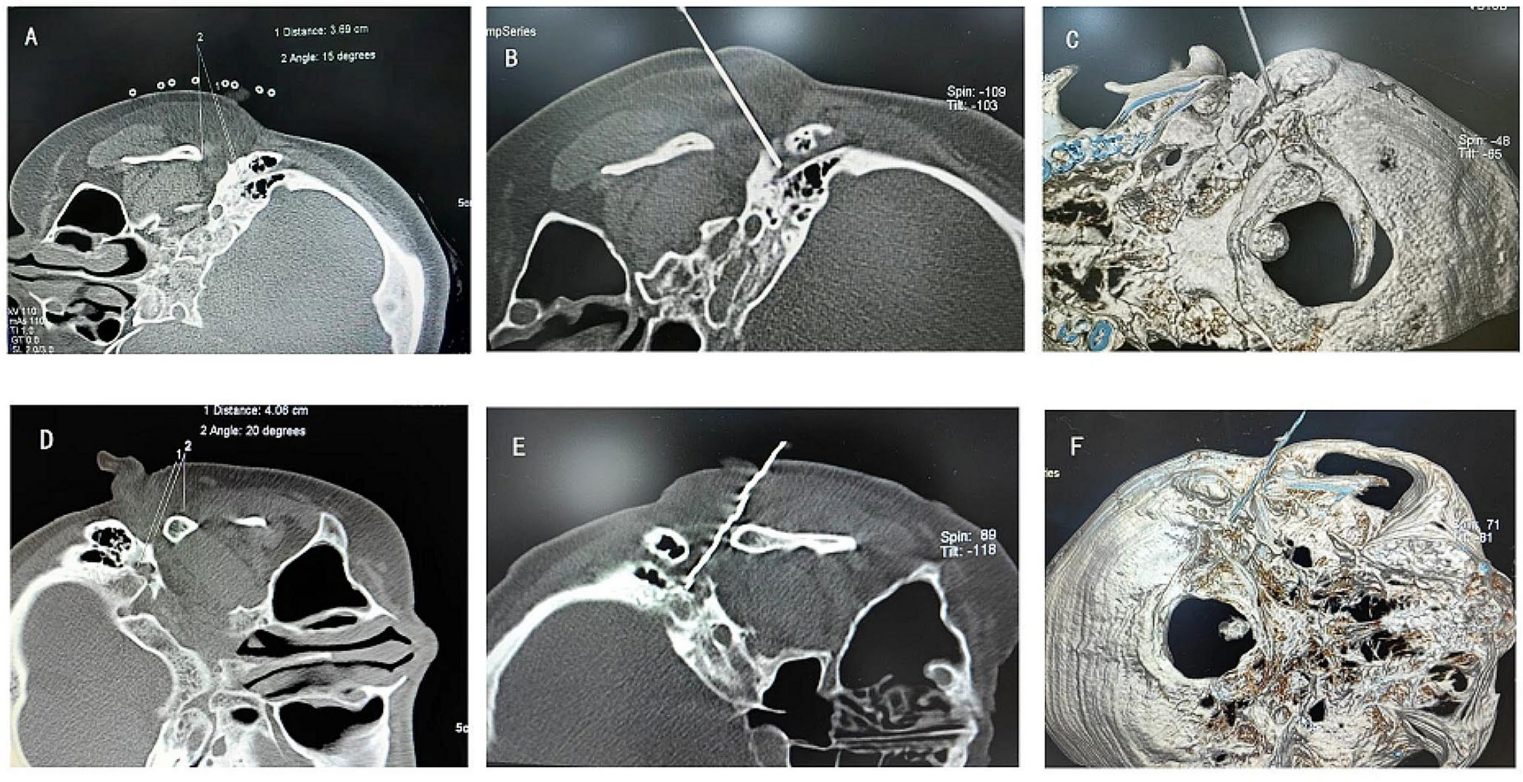
CT-guided radiofrequency ablation of facial nerve by bilateral stylomastoid foramen puncture. (**A**) Right stylomastoid foramen puncture path design and puncture parameters (depth, angle) measurement; (**B**) Right stylomastoid foramen puncture was successful; (**C**) CT three-dimensional reconstruction image after successful right stylomastoid foramen puncture; **D**, **E** and **F** are the left side

CT-guided radiofrequency ablation of the facial nerve: The patient initially lied on the left side of the CT table. A CT plane containing the right stylomastoid foramen was selected, and the puncture path was designed on this plane. The puncture was made to the target with a blunt radiofrequency needle under the guidance of CT, which was confirmed by a three-dimensional reconstruction of CT scans (Fig. 1A-C). This is followed by a motor nerve stimulation test with a low-frequency (2 Hz) current. Stimulation at 0.5-1 mA induces twitching in the right facial muscle at the same frequency as electrical stimulation. The patient was given continuous radiofrequency ablation at 65 °C for 30 s; at the same time, the patient was instructed to drum up their cheeks and close their eyes. Any indication of air leakage in the cheeks and inability to tightly close the right eye was observed and if noted, the radiofrequency was stopped immediately and the operation was terminated. If the right eye can be still closed tightly, the temperature is raised by 5 °C and the next cycle of radiofrequency is performed. Subsequently, the left side was treated in the same way (Fig. 1D-F).

**Fig. 2:**
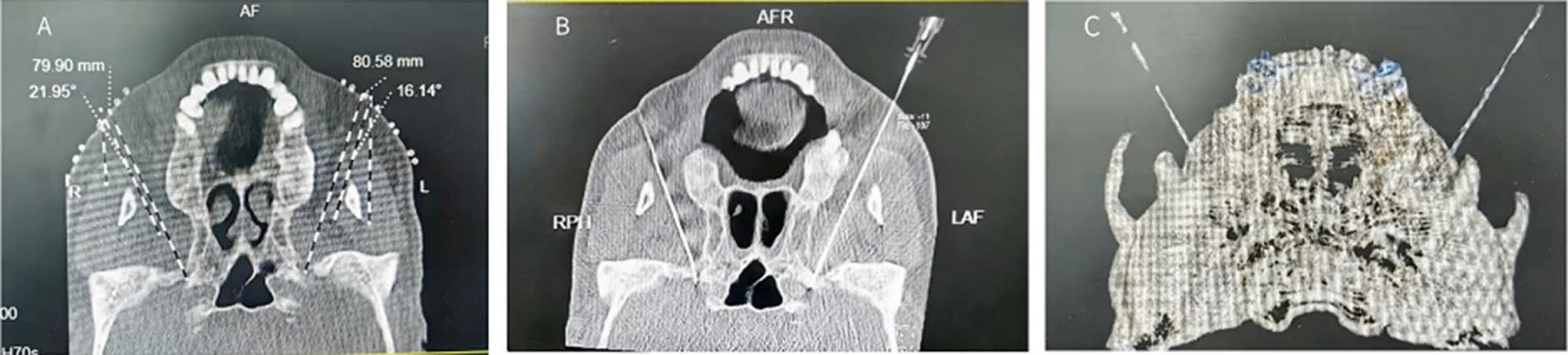
Radiofrequency ablation of mandibular branch of trigeminal nerve by bilateral foramen ovale puncture under CT guidance. (**A**) The puncture path design of bilateral foramen ovale. The puncture depth and angle of the patient’s left and right sides are 7.99 cm, 8.05 cm, 16.14° and 21.95° respectively. (**B**) the successful image of bilateral foramen ovale puncture, the puncture tip is flat on the inner orifice of foramen ovale and does not enter the skull; (**C**) CT three-dimensional reconstruction image after successful puncture of bilateral foramen ovale, with bilateral RF needle points located in the foramen ovale

CT-guided radiofrequency ablation of the mandibular branch of the trigeminal nerve: followed the method we published [6, 7], (Fig. 2A-C). After sensory and motor testing of the mandibular branch of the trigeminal nerve, standard radiofrequency ablation with step heating at 65–95 °C for 60 s is performed until the mandibular branch innervation area was numb and the mandibular twist disappears.

## Results

There were 106 patients diagnosed with Meige’s syndrome who accepted CT-guided radiofrequency ablation. Based on the inclusion and exclusion criteria, 6 patients (2 males and 4 females, aged 55–70 years) were enrolled. Their medical history ranged from 1 to 5 years.

All 6 patients received CT-guided percutaneous radiofrequency treatment of bilateral facial nerve within the stylomastoid foramen. The temperature was 65–90(76.67 ± 7.49)°C that radiofrequency treatment reaches the end standard. After 2–6 months of discharge observation, these patients were hospitalized again and received CT-guided percutaneous puncture of the mandibular branch of the trigeminal nerve in bilateral foramen ovale. When the radiofrequency treatment reached the end standard, the temperature was 70–85 °C (77.50 ± 4.52)°C.

After radiofrequency ablation of the facial nerve, the eyelid spasm and mouth twitch disappeared completely, and the bilateral mouth twitch was no longer skewed. However, all 6 patients experienced bilateral mild to moderate facial paralysis. Following the CT-guided radiofrequency puncture of the mandibular branch of the trigeminal nerve in the foramen ovale, the masticatory muscle spasm disappeared immediately, and the patients experienced no difficulty in opening their mouths. However, their bite force and chewing ability decreased. All patients reported hypoesthesia and numbness in the skin areas innervated by the bilateral mandibular nerves. (Refer to the video ).

All patients received follow-up in 4–28 months. The symptoms of mild facial paralysis in 6 patients disappeared within 2–5 (3.17 ± 0.94) months. Numbness in the mandibular branch of the trigeminal nerve and the decrease in sensation and chewing ability were improved, but they did not fully recover during the follow-up period.

## Discussion

Although the incidence of Meige’s syndrome is extremely low, it remains a challenging issue for clinicians because of its unknown pathogenesis [8]. Different types of Meige’s syndrome involve different cranial nerves [9, 10]. The conduction system of blepharospasm combined with oromandibular spasm is composed of the facial nerve and mandibular branch of the trigeminal nerve. Therefore, the combined radiofrequency ablation of the facial nerve and mandibular branch of the trigeminal nerve can block the transmission of abnormal motion signals to orbicularis oculi, orbicularis oris, and masticatory muscles along the facial nerve and mandibular nerve at the same time. Thus eliminating eyelid spasms and masticatory muscle spasms and achieving therapeutic relief for compound Meige’s syndrome.

The radiofrequency ablation of cranial nerve used in this treatment is a partial radiofrequency ablation to treat Meige’s syndrome while preserving part of the motor function of facial muscles. The results of this study showed that mild or moderate facial paralysis occurred after partial radiofrequency ablation of bilateral facial nerves. After 2–5 months, these facial paralysis symptoms improved and even disappeared. Although there was no difficulty in opening the mouth after radiofrequency ablation for partial mandibular branches of the bilateral trigeminal nerve, the remaining nerve had sensation loss and masticatory fatigue, which lasted for a long time. This requires that the radiofrequency parameters should be controlled more precise during radiofrequency ablation of mandibular branches of the trigeminal nerve further reduce the interference on masticatory ability.

The limitations of this study are that the sample size is indeed small, and the duration of postoperative follow-up time is not sufficient. The efficacy of combined radiofrequency ablation of the facial nerve and mandibular branch of the trigeminal nerve still needs long-term clinical observation in a multicenter large sample.

## Electronic supplementary material

Below is the link to the electronic supplementary material.

Supplementary Material 1

## Data Availability

All data generated and analyzed during the current study will be available from the corresponding author on reasonable request.
